# Genetic Mapping of QTL Associated with 100-Kernel Weight Using a DH Population in Maize

**DOI:** 10.3390/plants14121737

**Published:** 2025-06-06

**Authors:** Huawei Li, Hao Li, Jian Chen, Xiangbo Zhang, Baobao Wang, Shujun Zhi, Haiying Guan, Weibin Song, Jinsheng Lai, Haiming Zhao, Rixin Gao

**Affiliations:** 1 Crop Research Institute, Shandong Academy of Agricultural Sciences, Jinan 250100, China; lily984411@126.com; 2 School of Agriculture and Biology, Shanghai Jiao Tong University, Shanghai 200240, China; 3 State Key Laboratory of Plant Physiology and Biochemistry, Department of Plant Genetics and Breeding, China Agricultural University, Beijing 100193, China; 4 National Maize Improvement Center, Department of Plant Genetics and Breeding, China Agricultural University, Beijing 100193, China; 5 Guangdong Sugarcane Genetic Improvement Engineering Center, Institute of Bioengineering, Guangdong Academy of Sciences, Guangzhou 510316, China; 6 Biotechnology Research Institute, Chinese Academy of Agricultural Sciences, Beijing 100081, China; 7 Maize Research Institute, Shandong Academy of Agricultural Sciences, Jinan 250100, China

**Keywords:** maize (*Zea mays* L.), QTL mapping, 100-kernel weight, DH population

## Abstract

Grain yield establishment is a complex progress and the genetic basis of one of the most important yield components, 100-kernel weight, remains largely unknown. Here, we employed a double haploid (DH) population containing 477 lines which was developed from a cross of two maize elite inbred lines, PHBA6 and Chang7-2, to identify quantitative trait loci (QTL) that related to 100-kernel weight. The phenotypes of the DH population were acquired over three years in two different locations, while the DH lines were genotyped by next-generation sequencing technology of massively parallel 3ʹ end RNA sequencing (MP3RNA-seq). Eventually, 28,874 SNPs from 436 DH lines were preserved after SNP calling and filtering and a genetic map with a length of 837 cM was constructed. Then, single environment QTL analysis was performed using the R/qtl program, and it was found that a total of 17 QTLs related to 100-kernel weight were identified and distributed across the whole genome except chromosomes 5 and 6. The total phenotypic variation explained by QTLs detected in three different environments (BJ2016, BJ2107, and HN2018) was 22.2%, 32.9%, and 51.38%, respectively. Among these QTLs, three of them were identified across different environments as environmentally stable QTLs and explained more than 10% of the phenotypic variance each. Together, the results provided in this study preliminarily revealed the genetic basis of 100-kernel weight and will enhance molecular breeding for key agronomic kernel-related traits in maize.

## 1. Introduction

Maize is one of the most widely planted crops in the modern world. It has consistently been provided as food, livestock nutrition, and a resource of clean energy for decades [1]. According to FAOSTAT (https://www.fao.org/faostat/en/#data/QCL, accessed on 30 May 2025), maize production in China was 289,086,202 tons and the planting area was 44,256,978 ha in 2023, which were both the largest in cultivated crops in China. Maize plays a significant role in achieving global food security, and breeders are committed to breeding new maize varieties to meet changing production demands. With the help of the rapid development of molecular biology and biotechnology, our understanding of maize biology has been continuously deepened [2,3]. More and more studies have revealed the genetic mechanisms of vital agronomic traits and focused on genomic features and molecular genetics [4,5]. The application of the results from previous research has greatly improved the efficiency of genetic improvement and breeding in maize, which has tremendously contributed to the increase in grain yield and benefited human society [6,7].

Yield is a complex trait and it can be influenced by the biological nature of crops and environmental external factors [8,9]. It is found that most yield-related traits are regulated and affected by the joint action of quantitative trait loci (QTL) which often harbors many minor-effect genes [10]. By using technical means of traditional population genetics, numerous studies were conducted for QTL mapping and analysis based on different kinds of crops [5,11,12]. With the rise of next-generation sequencing and high-resolution genotype identification technologies, dissecting the genetic basis of important agronomic traits become much more accurate and properly easier [13]. Multiple genetic populations, such as traditional recombination inbred line (RIL) population, F_2_ population, and backcross (BC) population, as well as nested association mapping (NAM) panel, multiparent advanced generation inter-cross (MAGIC) panel, and other newly developed populations used in genome-wide association studies (GWAS), were cleverly constructed and applied to map-based deserting of genetic basis in plants [14,15,16,17,18]. By using all sorts of maize populations, plenty of QTLs associated with agronomic traits have been identified [19,20].

One of the most significant characteristics during the formation of crop yield is grain weight, also referred to as kernel weight. In the maize database MaizeGDB (http://www.maizegdb.org, accessed on 10 May 2025), there are 163 QTLs which relate to kernel weight in maize to date. These chromosomal regions can serve as potential targets for fine-mapping and gene cloning for future molecular breeding. Yet very few 100-kernel weight (HKW) QTLs have been properly cloned due to the genetic complexity of regulating mechanisms and interaction between minor-effect genes [21]. Several major QTLs of maize kernel-related traits were fine-mapped based on diverse genetic backgrounds and some vital candidate genes were identified within these QTL intervals in recent studies [22,23]. For instance, Li et al. [24] had fine-mapped a major QTL, *qhkw5*-*3*, for 100-kernel weight and eventually narrowed it down to a relatively short interval of 125 kb on chromosome 5 which harbors six candidate genes. And via a large BC_6_F_2_ segregation population, *qGW1.05* was fine-mapped to a 1.11 Mb interval [25]. These studies have demonstrated the effective power of QTL mapping for exploring the genetic basis and mining of the crucial genes controlling key kernel-related traits in maize. Also, some kernel mutants have been used as ideal materials for studying maize kernel traits, and a rich resource of genes involved in the development of seeds has been cloned and functionally characterized [26,27].

Nonetheless, the genetic mechanism underlying maize yield-related traits remains largely unknown, and it urgently needs to be further studied as today’s fast improvement and achievements from genetics and genomics were made in plants [28]. Accurate phenotypic and high-quality genotypic data are the insurance of reliable QTL mapping research [29]. An economical and practical massively parallel 3ʹ end RNA sequencing (MP3RNA-seq) method was reported by our group which only cost one-tenth compared to traditional RNA-seq [30]. And the function of genotyping of MP3RNA-seq was demonstrated by identifying eQTLs and classic QTLs excellently for plant height and ear height using a double haploid (DH) population consisting of 477 lines which was constructed using 2 elite inbred lines, PHBA6 and C7-2 [30]. Among traditional linkage populations and modern association panels, DH populations were widely applied for breeding programs because they were equipped with the advantages of nice repeatability in phenotype collection and effectively rapid construction procedure which allow us to conduct experiments more accurately and comprehensively, and better evaluate the results [30]. In order to investigate the genetic basis of maize 100-kernel weight, the authors evaluated the phenotype in different environments and performed QTL mapping and analysis based on the same DH population. Our QTL analysis showed that a total of seventeen HKW QTLs were detected across three different environments, three of which could explain over 10% of the phenotypic variance each. Together, this study will broaden our vision about the genetic mechanism of 100-kernel weight, lay a solid foundation for cloning major QTLs, and provide theoretical support for maize molecular breeding.

## 2. Results

### 2.1. Phenotypic Variations of 100-Kernel Weight in DH Population

**Figure 1:**
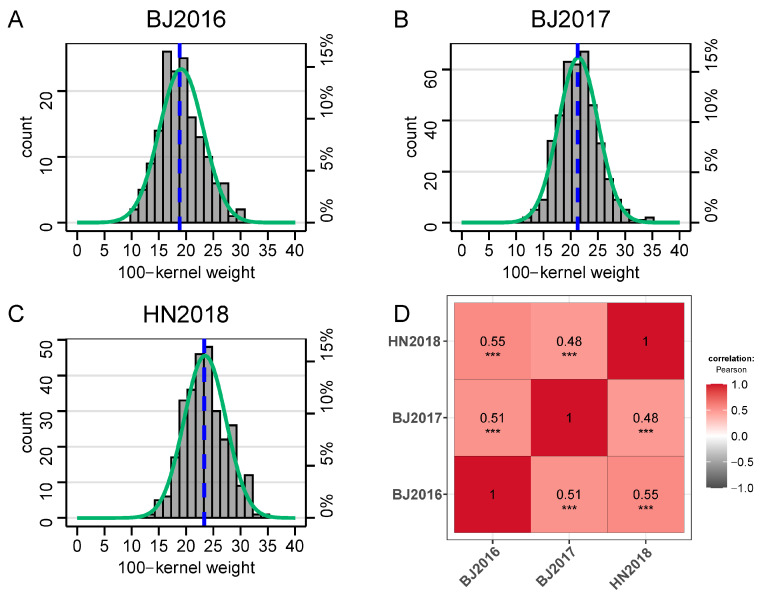
The performance of 100-kernel weight in the DH population and the heatmap of correlation analysis for different environments. (**A**–**C**), the distribution of 100-kernel weight in different environments. Green curve represents simulated normalization curve. Blue dot line marks the mean value in this environment. (**D**), correlation heatmap of 100-kernel weight obtained from different environments. The color depth represents Pearson correlation coefficient. *** *p* < 0.001.

DH family lines were harvested and five ears per plot were collected for measuring 100-kernel weight. We found that the field performances of 100-kernel weight in the DH population across three different environments exhibited relatively large variance (Table 1). Also, the variance of each environment exceeded the performance of the parents (Figure S1), showing that the DH population contained enough recombinant events and a complex mechanism under an important agronomic trait. HKW of the DH population varied from 10.5 (BJ2016) to 34.5 g (BJ2017) across different environments (Table 1). Mean values of HKW in BJ2016, BJ2017, and HN2018 were 19.1, 21.4, and 23.5 g, respectively, the performance of which in BJ2016 was less than 20 g, showing that the weather conditions in that environment may affect the pollination efficiency or accumulation of dry matter in grains. The Shapiro–Wilk test showed that HKW in BJ2016, BJ2017, and HN2018 all exhibited a normal distribution (Figure 1A–C). The coefficients of variation (CV) in the DH population were up to 21.2% in different environments (Table 1) and slightly higher compared to the statistical analysis of the same trait in our previous study [31]. Phenotypic correlation coefficients of HKW among three environments were ~0.50 and significant at the level of *p* < 0.001 in the DH population (Figure 1D). At last, the BLUP values of HKW based on three environments were estimated and it showed that the BLUP dataset followed a normal distribution (Figure S2).

### 2.2. Construction of the Genetic Linkage Map

**Figure 2:**
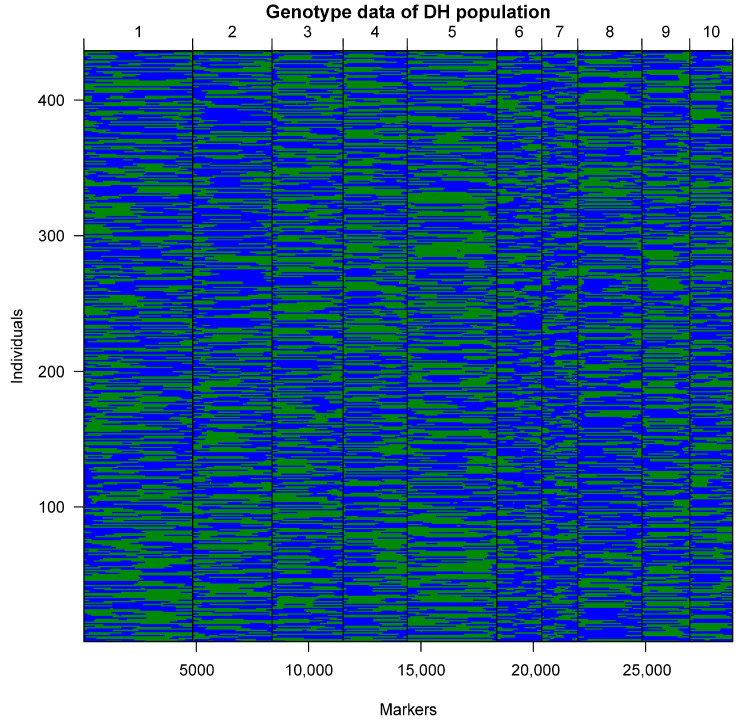
Genotype data and genetic map construction of the DH population. The genetic linkage map of DH lines is shown as follows blue and green dots represent the genotypes of PHBA6 and Chang7-2, respectively.

The leaf tissues and stem under shoot apical meristem at the elongation stage of the parental lines of PHBA6 and C7-2, and DH lines were collected for later MP3RNA-sequencing. About 1.33 billion reads were acquired from sequencing and which were further mapped to the B73 reference genome. Next, SNP calling was performed and it resulted in 35,836 high-quality SNPs between PHBA6 and C7-2. These SNPs were distributed on all ten chromosomes and most of them (~85%) were annotated in the exons of genes (Figure S3). There were 8105 recombinant events detected by the analysis of genotypic blocks (Table S1). Finally, after the calling and the filtering of high-quality SNPs, 28,874 SNPs in 436 DH lines were used for the construction of a genetic linkage map which extended a genetic length of 837 cM calculated by using *qtl* package (Figure 2; Table S1). The average distance between molecular markers of these SNPs was 0.11 to 8.85 cM. The pair-wise recombination fractions and the LOD scores of the markers were checked and displayed no wrongdoing. And the SNP genetic map was used for further mapping and analysis.

### 2.3. QTL Mapping and Analysis for 100-Kernel Weight

**Figure 3:**
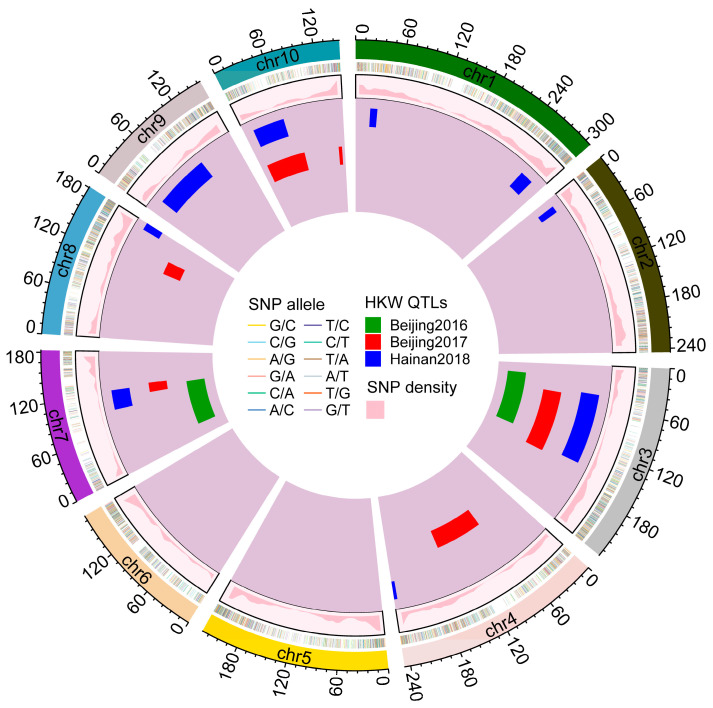
QTLs identified across different environments using the DH population. Track a, b, and c represent SNP allele, SNP density, and QTL intervals, respectively. Colored bars in track c showed QTLs in different environments as: green, Beijing2016; red, Beijing2017; blue, Hainan2018.

**Figure 4:**
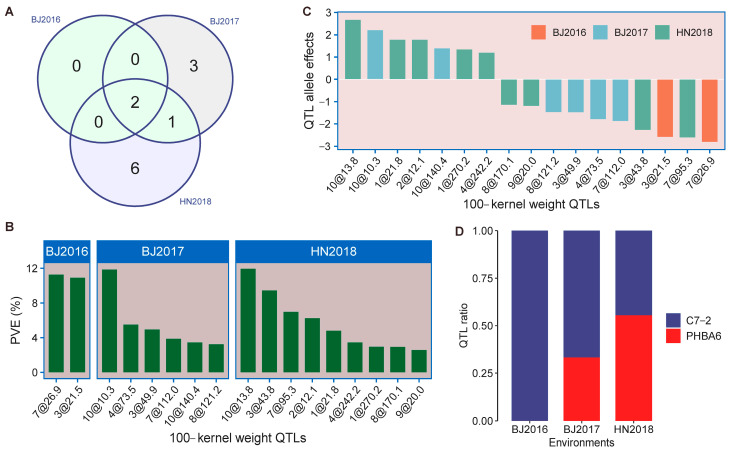
QTL distribution, QTL allele effects, and PVE in the DH population. (**A**) Venn plot showed QTL number distribution and overlapped QTLs across different environments. (**B**) Phenotypic variations explained by QTL were shown as colored bars. (**C**) allele effects of each QTL and (**D**) ratio of the identified QTLs across three environments.

By using the genetic map constructed of 436 DH lines and the phenotype obtained from three environments, we performed QTL mapping for HKW using single environment analysis. In total, seventeen QTLs associated with HKW were detected under the threshold of LOD score 3 (Figure 3). These QTLs were distributed on all the chromosomes except for chromosomes 5 and 6 (Figure 3). There were two, six, and nine QTLs identified in BJ2016, BJ2017, and HN2018, respectively (Figure 4A), and the corresponding total phenotypic variance explained (PVE) were 22.2%, 32.9%, and 51.4% (Table S2). The PVE of QTLs identified by single environment analysis varied from 2.6% to 12.0% (Figure 4B; Table S2). Also, we found that three among these QTLs were identified in multiple environments on chromosomes 3, 7, and 10, which was described as “environmentally stable QTL” in previous studies [32,33], and the contribution of each single stable QTL were all exceeded 10%, with a max LOD score up to 13.4 in BJ2017 (Figure 3; Table S2). It suggests that these three major QTLs may play a crucial role in the formation of 100-kernel weight and would be key target regions responsible for grain yield. Next, we conducted a combined analysis of different environments where the BLUP values were estimated and proceeded using the QTL mapping procedure based on the SNP genetic map. Eight QTLs related to HKW were detected, all of which overlapped with QTLs from single environment identification (Table S3). Additionally, the additive effects of the QTLs for HKW were estimated and it ranged from −2.8 to 2.7, showing that the ratio of PHBA6 alleles was lower than C7-2 alleles in these QTLs (Figure 4C,D; Table S2). Major QTLs on chromosomes 3 and 7 showed a negative effect, while the one on chromosome 10 had a positive effect, indicating that the increased effect for HKW from both parental alleles on different chromosomes might be working collaboratively. Finally, we took a glance at the genes harbored in an overlapped interval with a length of 9.2 Mb of the major QTL on chromosome 7 based on the B73 v4 reference genome. It was found that within this interval, a total of 321 protein-coding genes were included. We compared RNA-seq data on maize seed development dynamics in a previously published study [34] and found that among these genes, 73 were specifically expressed in seeds (Figure S4). Among these 73 genes, 53 were annotated, 11 of which were transcription factors. These results suggest the significant importance of analyzing the QTLs underlying the genetic basis of 100-kernel weight and it is of great complexity for the molecular mechanism of crop yield formation.

## 3. Discussion

To dissect the genetic basis of important agronomic traits, many previous studies conducted genetic research using a map-based strategy on different types of populations in maize [32,35,36]. A number of QTLs associated with yield-related traits were identified across genetic backgrounds [9,20,22]. Bi-parental populations and high-density genetic maps based on SNP markers are often used for QTL mapping, and a RIL population that consisted of 80 F_7:8_ lines was applied for QTL mapping of eight agronomic traits using 2904 SNP markers [20]. Whereas in our present study, a DH population contained more than 470 lines derived from the cross of elite inbred lines PHBA6 and C7-2 was used to analyze the genetic basis of 100-kernel weight, and the genetic linkage map was constructed by nearly ten times SNP markers of which in their RIL population. The DH population is one of the most ideal traditional mapping populations for its efficient and effective construction and research related to maize biology was conducted relying on DH populations [37,38]. Yang et al. [39] evaluated a set of DH lines from the most spread hybrid in China, Zhengdan958, and performed QTL analysis on yield components. Six ear traits, including HKW, were investigated and the QTLs related to HKW identified from single environment analysis on chromosomes 3, 7, and 10 overlapped with our present study. However, the QTLs on chromosomes 3, 7, and 10 from their study were detected only in one single environment, and within the DH population in our study, those were detected across different multi-environments (Figure 3). Noted, the paternal line is C7-2 which is the same between their population and ours, and perhaps it can explain why there are a lot of overlapped genomic regions in the QTLs identified between Yang’s study and ours. The additive effect alleles from the major QTL identified on chromosome 7 in our study were from C7-2 which were also observed in their study [39]. These results showed the feasibility of QTL mapping using DH populations, and it indicates that the major QTL on chromosome 7 will be a key target region to reveal the potential mechanism underlying 100-kernel weight and worth further studying in the future.

In the past two decades, sequencing technologies have made significant progress [40]. With the support of sequencing development, we were able to dig deeper into the genomes and identify more genomic variation in important crops [2,31,41,42]. Several previous studies have proven the power of a large population and high-density genetic linkage maps for QTL mapping and discovering critical candidate genes [10,43,44]. Also, previous studies have shown that employing methods such as association analysis would enhance discovering genetic variation and mining important candidate genes [45,46]. A combination of GWAS and meta-QTL analyses have located six candidate genes related to kernel weight and width using 1283 maize inbred lines, where in our study, two of which were near the QTLs on chromosomes 1 and 3, but none overlapped [45]. Another study for 100-kernel weight showed that five significant SNPs around 122 Mb were identified using a GWAS panel of 200 maize inbred lines, which overlapped with the major QTL detected across three environments on chromosome 3 in our present study, suggesting that this QTL could be key target loci and resource for further studies and breeding for maize [46]. Among GWAS studies and high-density map projects, the sequencing costs of parental and family lines in populations were usually fairly high. MP3RNA-seq allows us to theoretically sequence 96 samples within one library using two levels of unique barcodes to distinguish different samples, and it is very effective for calling SNPs and genotyping [30]. In the present study, an SNP genetic map was constructed and used for later QTL mapping for HKW. The average markers per DH line in this study were identified and it was far more than that in the traditional studies where PCR markers were applied [47]. By using the SNP map, we have identified several QTLs associated with 100-kernel weight, and meanwhile, a bin map was constructed by the “sliding window” method reported in previous papers [10] using the identified SNPs in our DH population to validate the results (Figure S5; Table S4). Then, the bin map and phenotypic BLUP values were used for QTL mapping and analysis for the trait HKW, and the results were similar to the outcome from the SNP map (Figure S6).

Up to this date, a lot of QTLs related to yield, including some overlapped QTLs in our present study, were investigated in previous studies in maize [48,49]. For instance, Li et al. [48] fine-mapped a major QTL responsible for kernel weight and width, and identified *GRMZM2G114706* as a candidate gene using a large BC_3_F_4_ segregating population, which was harbored in the major QTL detected on chromosome 7 in the present study. Yang et al. [49] reported a genome assembly of an inbred line named SK, and performed map-based cloning of a QTL, *qHKW1*, which overlapped with a QTL identified on chromosome 1 in HN2018 by our DH population with a positive additive effect (Figure 3 and Figure 4C; Table S2). This work marks the cloning of the first maize kernel weight (KW) QTL, *ZmBAM1d*, which was targeted for selection during maize improvement [49,50]. Kernel weight is an important agronomic trait in modern maize breeding, and the contribution of KW improvements to yield genetic gain was smaller but substantial, compared with other kernel-related traits like kernel number (KN), and crop improvement had conferred on modern hybrids greater KW plasticity [51]. Nonetheless, researchers also observed potential improvements in individual KW in Chinese hybrids that remain unexploited because KN was the fundamental target trait to increase grain yield [51]. So, the superior large grain genotypes possibly have much potential in the future breeding of maize. In our study, QTLs associated with 100-kernel weight were discovered in a DH population derived from PHBA6 and C7-2, the genomic intervals of which were relatively wide, limiting their application in breeding. But with future development of functional molecular markers based on the DH population, fine-mapping of these major QTLs, and the cloning of crucial candidate genes, we believe these findings will benefit modern breeding and the course of improvement in maize. In the meantime, modern breeding technologies, such as marker-assisted recurrent selection (MARS), the transgenic approach, genome editing (GE), and doubled haploid (DH) technology, to shorten the breeding cycle time are helpful and essential for the application of the results in our study and so forth [52].

In addition, pleiotropic effects were observed in the QTLs identified using this DH population in the present study. For example, the QTL on chromosome 3 was overlapping with a QTL that controlled leaf angle which was also detected across multiple environments reported by Zhu et al. [36], implying the complexity of the genetic mechanisms of 100-kernel weight. These previously identified co-located QTLs in other studies have proven the reliability of the genetic map and phenotypes used in this study, and the QTL confidential intervals identified in this study are needed for further exploration for more understanding of maize kernel weight formation. Despite the exciting results acquired from this study, we have noticed some small drawbacks such as the relatively less phenotypic dataset collected from the environment BJ2016, and the inability to analyze the dominance effect for the QTLs since the DH lines are pure lines. There were 158 DH lines evaluated in BJ2016, and it showed that the phenotype data in BJ2016 accorded with normal distribution. However, the single environment QTL mapping in BJ2016 resulted in only two QTLs which were less compared with the other two environments that contained more DH lines, indicating that fewer family lines in populations might affect detecting power when QTL mapping was performed. Moreover, whether the specific genetic background of these QTLs would influence how they can be used with different parents needs to be validated in the future. Among the QTLs detected in our study, we noticed that some of them were minor-effect QTLs, indicating the great genetic complexity of the agronomic traits. During the maize breeding process, the better choice for materials selection will need larger populations to ensure enough variations. Overall, the results from this research have shown us several highly credentialed genomic regions associated with yield-related traits in maize, and provide more target loci for underlying the basis of 100-kernel weight which could be further studied and eventually be beneficial for future molecular breeding.

## 4. Materials and Methods

### 4.1. Plant Materials and Phenotyping

The population used in the present study was constructed by 477 DH lines derived from a hybrid of two parental inbred lines, PHBA6 and C7-2, which was reported in our previous study [30]. PHBA6 and C7-2 are two elite inbred lines which have been widely used for modern maize improvement and breeding, and these two inbred lines both have excellent performance in production and stress resistance. Based on previous field observation for yield-related traits between PHBA6 and C7-2, we have noticed that 100-kernel weights were significantly different; hence, these two inbred lines were ideal materials for studying 100-kernel weight. For the development of DH lines, firstly, the F1 hybrids of PHBA6 and C7-2 were crossed with a maize haploid inducer CAU3 in 2014 in Sanya. The putative haploid kernels were selected and planted at the field nursery during the summer of the year 2015 in Shangzhuang. The putative haploids were further screened using the features of shorter stature and smaller biomass in the field. Approximately 1% of the haploid plants were successfully self-crossed by the natural doubling method and finally produced the DH population used in this study [36]. The DH population was planted at two locations (Hainan, Yazhou, 109°06′ E, 18°35′ N; Beijing, Shangzhuang, 116°10′ E, 40°13′ N) for three years (2016, 2017 and 2018). The materials were planted on 27 April 2016 and 30 April 2017 in Beijing, and 3 November 2018 in Hainan, respectively (Table S5). With a 3 m long and 0.5 m wide one-row block, a randomized complete block design was applied for material planting in trial fields. Each DH line was planted with a plant distance of 0.25 m in one plot containing 17 plants with two biological replicates. Field experiments were under regular management and strict artificial self-bred was conducted during the pollen shedding period. Then, five ears per plot for each DH line were harvested and 100-kernel weight was measured to collect the phenotypic data. Due to the continuous rain and waterlogging damage in 2016, the seed setting rate of some DH lines was low and we had those cast out, and a relatively smaller dataset of BJ2016 was acquired and used for phenotypic analysis. The broad-sense heritability (*h*^2^) of 100-kernel weight was calculated as described in previous studies [53]. Meanwhile, we estimated the best linear unbiased predictions (BLUPs) of all environments and repetitions using the method of maximum likelihood by R/lme4 (version 1.1-33) software [53].

### 4.2. Genetic Linkage Map Construction

The leaf tissues of the DH population and two parental lines were collected for MP3RNA-seq as described in our previous study [30]. Total RNA was extracted and processed following the protocol of the MP3RNA-seq method. The libraries constructed for RNA-seq were sent to sequence on an Illumina X Ten platform. Raw reads from sequencing were then mapped to B73 reference genome V4 using Hisat2 (version 2.0.4), and single nucleotide polymorphism (SNP) calling was performed using SAMtools (version 0.1.16) and BCFtools (version 0.1.16). As a result, 35,846 SNPs were identified and an average of 19,907 SNPs were detected among DH lines [30]. In addition, the SNPs with partial segregation greater than 2/1 (Chi-square test, *p* < 1.0 × 10^−7^) or a heterozygosity rate greater than 15% were discarded, and the DH lines with a heterozygosity rate greater than 15% were also eliminated. Finally, 436 DH lines were retained for further genetic map construction and QTL mapping. For genetic linkage map construction, we first used the *argmax* command in R/qtl (version 1.44-9) software to impute the missing genotypes. Then, the command of *est.map* was used for map construction and the linkage calculation method was using “*kosambi*” [10].

### 4.3. QTL Mapping and Analysis

The method of composite interval mapping (CIM) which was often adopted in identifying QTLs was applied in the present study for QTL mapping for 100-kernel weight [54]. It was conducted by using the R/qtl package [10]. The threshold value of the logarithm of odds (LOD) was determined with a score of three based on the commonly used cutoff in several previous studies to declare significant QTLs [31,55,56]. The confidential interval of each QTL was defined by the 1.5 LOD-drop method [10]. And the addictive effect and phenotypic variance explained (PVE) by each QTL were estimated by linear models using R function *lm* to achieve [36].

## Figures and Tables

**Table 1:** The phenotypic performance of 100-kernel weight of the parents and the DH population.

	Env. ^a^	Mean ± SD (g)	Range (g)	Kurtosis	Skew	CV ^b^ (%)	*h*_2_ ^c^ (%)
Population	BJ2016	19.12 ± 4.05	10.45–30.52	0.03	0.41	21.17	79.70
	BJ2017	21.40 ± 3.64	11.60–34.50	0.49	0.36	17.02	
	HN2018	23.47 ± 3.85	13.86–33.87	−0.38	0.17	16.39	
	BLUP	21.07 ± 1.88	16.06–28.83	0.86	0.37	8.91	
	Overall	21.49 ± 3.53	10.45–34.50	0.74	0.35	16.43	
C7-2	BJ2016	26.31 ± 3.22					
	BJ2017	29.10 ± 2.67					
	HN2018	28.71 ± 3.00					
PHBA6	BJ2016	18.53 ± 4.04					
	BJ2017	20.91 ± 2.51					
	HN2018	21.12 ± 3.62					

^a^ Environments. ^b^ Coefficient of variation. ^c^ Broad-sense heritability.

## Data Availability

The data sets generated in this study are presented in the paper and can be found in the National Center for Biotechnology Information Sequence Read Archive (http://www.ncbi.nlm.nih.gov/sra, accessed on 28 April 2025) under accession number SRP149505.

## Supplementary Materials

The following supporting information can be downloaded at https://www.mdpi.com/article/10.3390/plants14121737/s1, Figure S1: 100-kernel weight across three environments between parental lines and the DH population; Figure S2: Distribution of the BLUPs estimated from different environments for 100-kernel weight in the DH population; Figure S3: SNP distribution in the DH population; Figure S4: The relative expression of candidate genes showed by the heatmap of 0 to 38 DAP seeds in the overlapped interval of the environmentally stable QTL identified on chromosome 7 in the DH lines; Figure S5: Bin map constructed by the DH population; Figure S6: QTL mapping for the BLUPs using the bin map; Table S1: Information of SNP map; Table S2: Profile of the QTLs associated with HKW identified in this study; Table S3: The profile of QTLs identified using the BLUP values of the DH population in this study; Table S4: The bin map constructed by using the SNPs identified in the DH population; Table S5: The information of the environments.

## Abbreviations

The following abbreviations are used in this manuscript:

DH	Double Haploid
QTL	Quantitative Trait Loci
MP3RNA-seq	Massively Parallel 3′ end RNA sequencing
SNP	Single Nucleotide Polymorphism
HKW	100-Kernel Weight
RIL	recombination inbred line
BC	BackCross
NAM panel	Nested Association Mapping panel
MAGIC	Multiparent Advanced Generation Inter-Cross
GWAS	Genome-Wide Association Studies
CV	Coefficient of Variation
LOD	Logarithm of the Odds
PVE	Phenotypic Variance Explained
BLUP	Best Linear Unbiased Prediction
CIM	Composite Interval Mapping
BJ2016	Beijing2016
BJ2017	Beijing2017
HN2018	Hainan2018
MARS	Marker-Assisted Recurrent Selection
GE	Genome Editing
KW	Kernel Weight
KN	Kernel Number
