# Supplementary material for: Genetic Mapping of QTL Associated with 100-Kernel Weight Using a DH Population in Maize
Source: Plants (Basel). 2025 Jun 6;14(12):1737. doi: 10.3390/plants14121737 (PMC12197198; doi:10.3390/plants14121737)
Supplement: Supplementary file 1 [file plants-14-01737-s001.zip › Supplementary_FigureS1-S6.pdf]

Title: Genetic mapping of QTL associated with 100-kernel weight using a DH population in maize

Huawei Li [1], Hao Li [2], Jian Chen [3], Xiangbo Zhang [4], Baobao Wang [5], Shujun Zhi [6], Haiying Guan [6], Weibin Song [3], Jinsheng Lai [3], Haiming Zhao [3], Rixin Gao [6]*

[1]*Crop Research Institute, Shandong Academy of Agricultural Sciences, Jinan 250100, China*

[2]*School of Agriculture and Biology, Shanghai Jiao Tong University, Shanghai 200240, China*

[3]*State Key Laboratory of Plant Physiology and Biochemistry and National Maize Improvement Center, Department of Plant Genetics and Breeding, China Agricultural University, Beijing, 100193, China*

[4]*Guangdong Sugarcane Genetic Improvement Engineering Center, Institute of Bioengineering, Guangdong Academy of Sciences, Guangzhou 510316, China*

[5]*Biotechnology Research Institute, Chinese Academy of Agricultural Sciences, Beijing 100081, China*

[6]*Maize Research Institute, Shandong Academy of Agricultural Sciences, Jinan 250100, Shandong Province, China*

**Address for corresponding author:*

Rixin Gao, Ph. D.

Maize Research Institute, Shandong Academy of Agricultural Sciences

No. 23788 Gongye North Rd, Licheng District, Jinan 250100

E-mail: one77@cau.edu.cn; gaorixin@saas.ac.cn

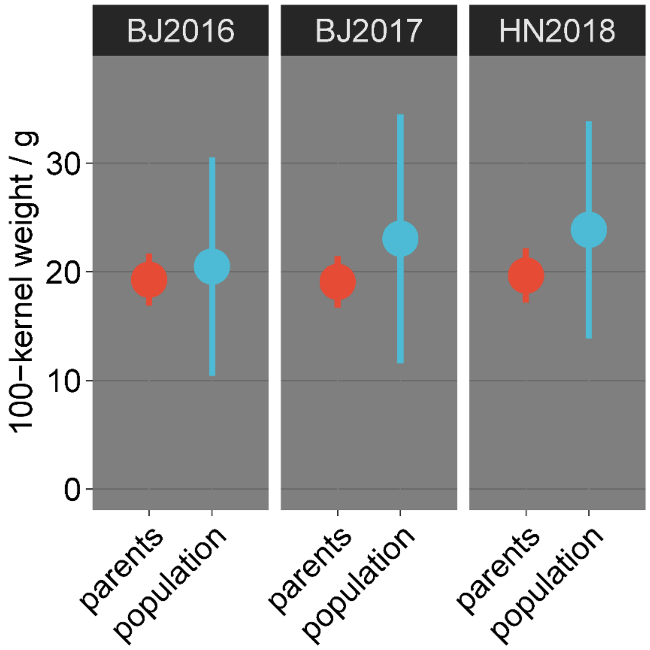

Figure S1. 100-kernel weight across three environments between parental lines and the DH population

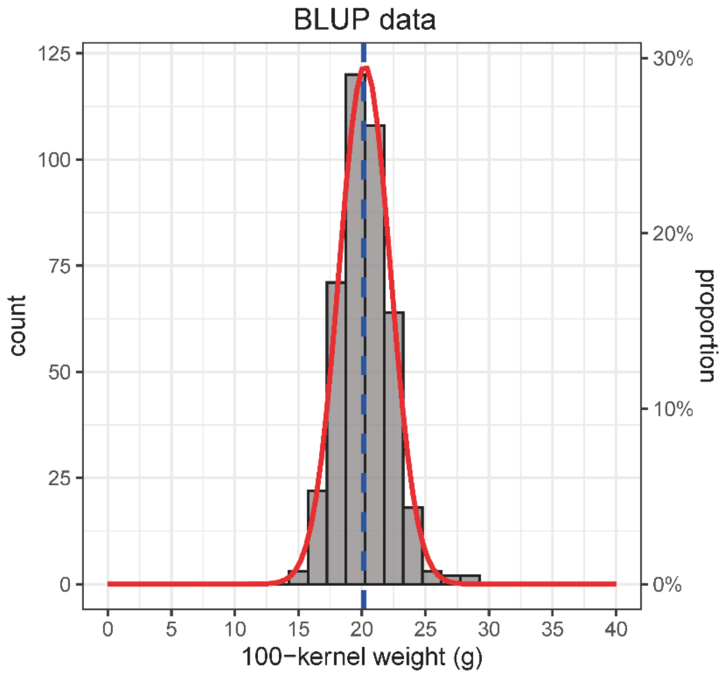

Figure S2. Distribution of the BLUPs estimated from different environments for 100-kernel weight in the DH population

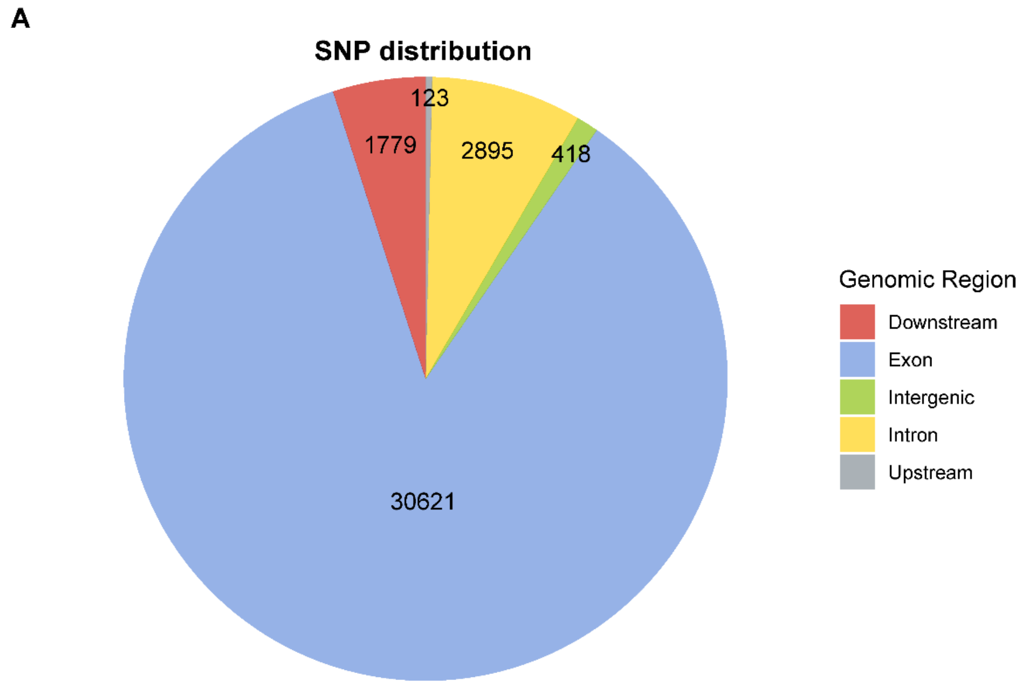

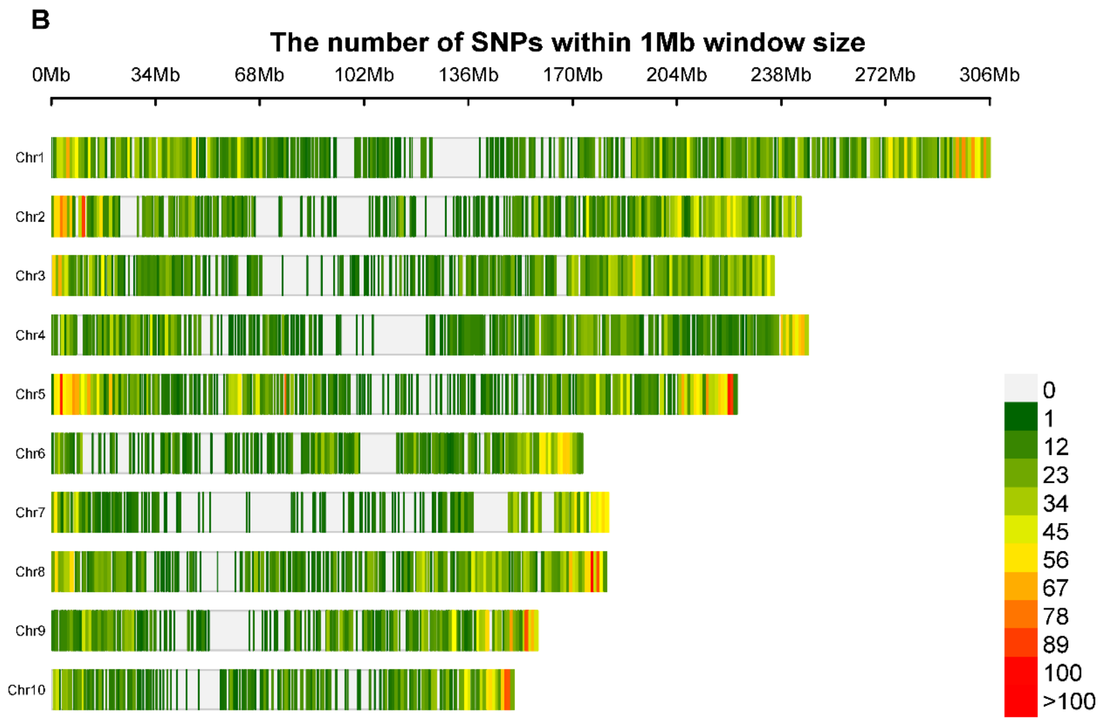

Figure S3. SNP distribution in the DH population. (A) The genomic regions where the SNP is located. (B) Heatmap of the number of SNPs within 1 Mb window across the genome.

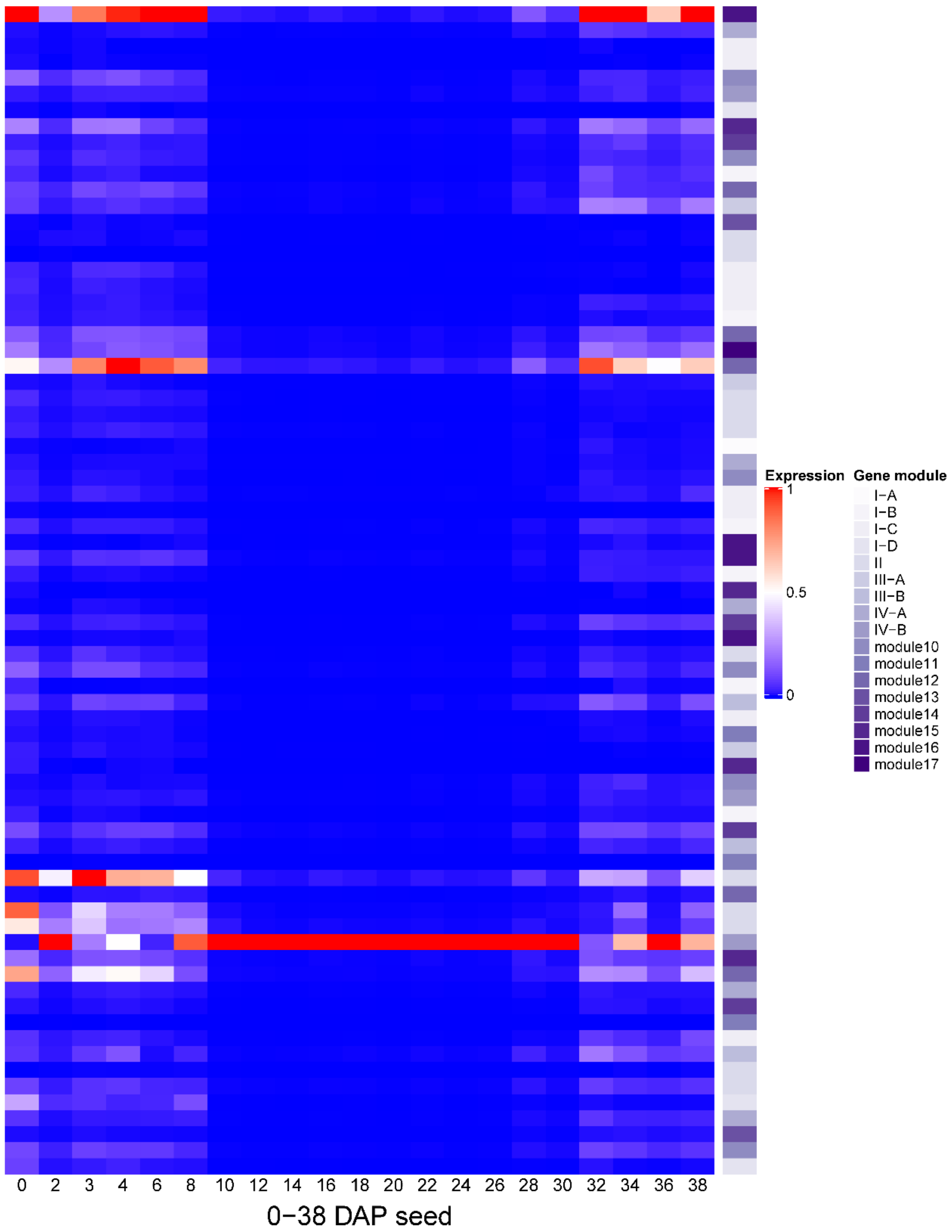

Figure S4. The relative expression of candidate genes showed by the heatmap of 0 to 38 DAP seeds in the overlapped interval of the environmentally stable QTL identified on chromosome 7 in the DH lines.

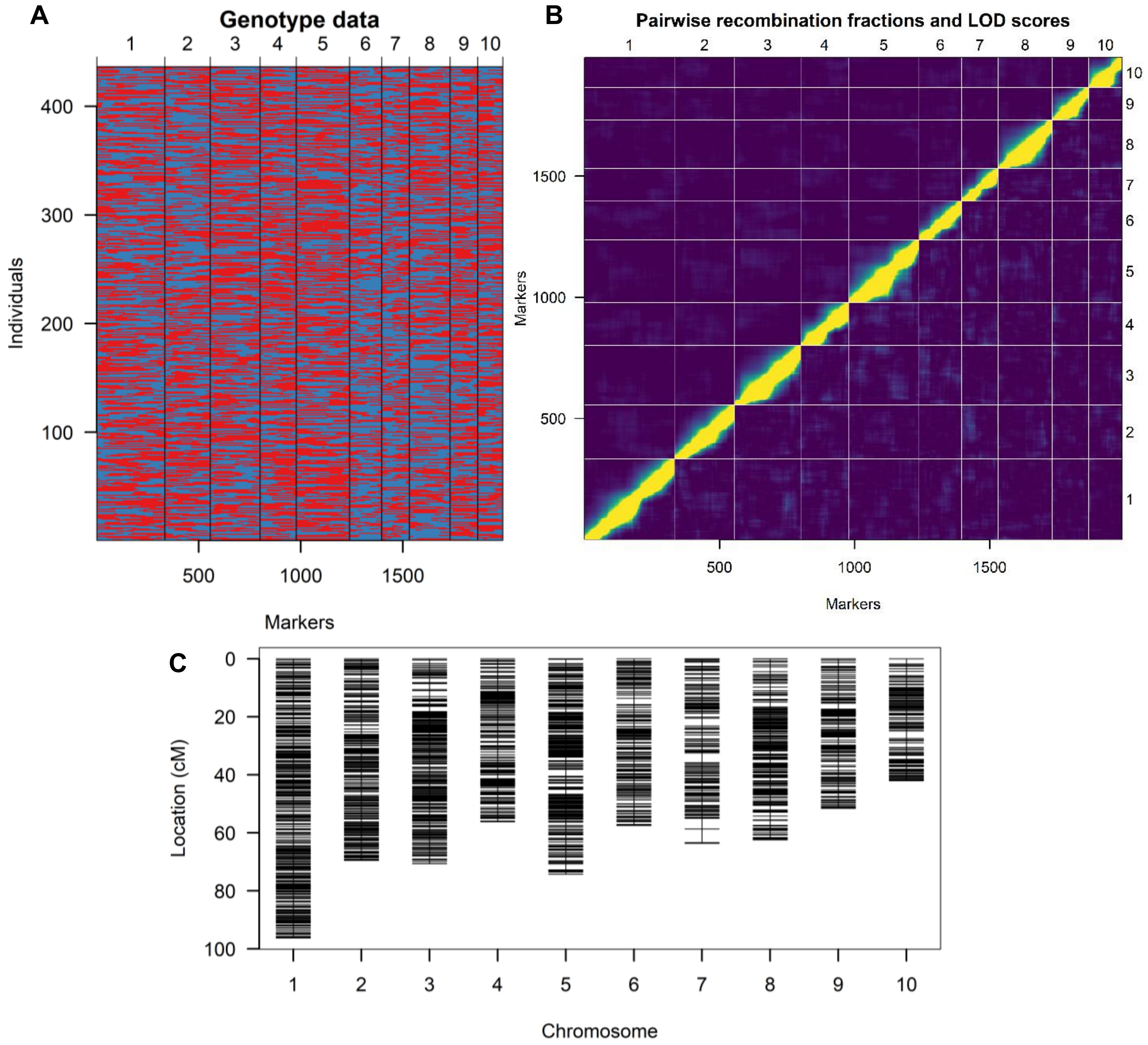

Figure S5. Bin map constructed by the DH population. (A) The genotypic information of DH lines. Red represents C7-2 genotype and blue represents PHBA6 genotype. (B) The pairwise analysis for recombination fractions (Left upper half) and the corresponding LOD scores (Right lower half) showed by heatmap when dark blue means lower values and yellow represents higher values. (C) Genetic linkage map of the bin markers and y axis marks genetic distance.

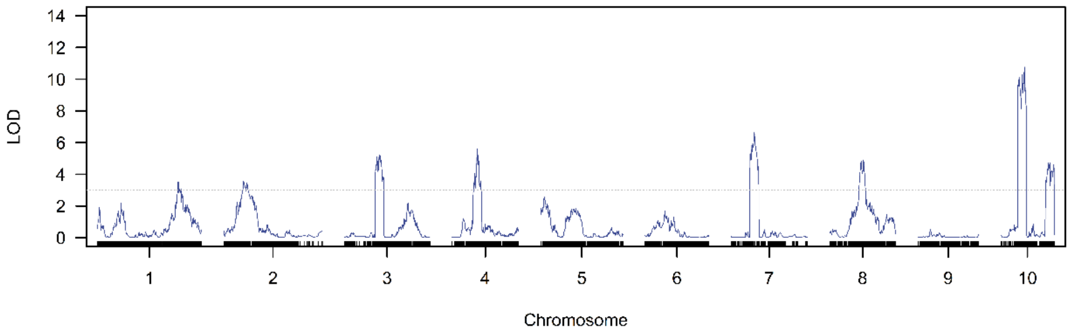

Figure S6. QTL mapping for the BLUPs using the bin map
